# Long non‐coding RNA (lncRNA) H19 induces hepatic steatosis through activating MLXIPL and mTORC1 networks in hepatocytes

**DOI:** 10.1111/jcmm.14818

**Published:** 2019-12-06

**Authors:** Hao Wang, Youde Cao, Liqing Shu, Ying Zhu, Qi Peng, Longke Ran, Jinghong Wu, Yetao Luo, Guowei Zuo, Jinyong Luo, Lan Zhou, Qiong Shi, Yaguang Weng, Ailong Huang, Tong‐Chuan He, Jiaming Fan

**Affiliations:** ^1^ Ministry of Education Key Laboratory of Diagnostic Medicine, and School of Laboratory Medicine Chongqing Medical University Chongqing China; ^2^ Molecular Oncology Laboratory Department of Orthopaedic Surgery and Rehabilitation Medicine The University of Chicago Medical Center Chicago IL USA; ^3^ Department of Pathology Chongqing Medical University Chongqing China; ^4^ Department of Bioinformatics Chongqing Medical University Chongqing China; ^5^ Department of Biostatistics School of Public Health and Management Chongqing Medical University Chongqing China; ^6^ Key Laboratory of Molecular Biology for Infectious Diseases of The Ministry of Education of China Department of Infectious Diseases Institute for Viral Hepatitis The Second Affiliated Hospital Chongqing Medical University Chongqing China

**Keywords:** hepatic lipid metabolism, lncRNA H19, MLXIPL, mTORC1, non‐alcoholic fatty liver disease

## Abstract

Liver plays an essential role in regulating lipid metabolism, and chronically disturbed hepatic metabolism may cause obesity and metabolic syndrome, which may lead to non‐alcoholic fatty liver disease (NAFLD). Increasing evidence indicates long non‐coding RNAs (lncRNAs) play an important role in energy metabolism. Here, we investigated the role of lncRNA H19 in hepatic lipid metabolism and its potential association with NAFLD. We found that H19 was up‐regulated in oleic acid‐induced steatosis and during the development of high‐fat diet (HFD)‐induced NAFLD. Exogenous overexpression of H19 in hepatocytes induced lipid accumulation and up‐regulated the expression of numerous genes involved in lipid synthesis, storage and breakdown, while silencing endogenous H19 led to a decreased lipid accumulation in hepatocytes. Mechanistically, H19 was shown to promote hepatic steatosis by up‐regulating lipogenic transcription factor MLXIPL. Silencing Mlxipl diminished H19‐induced lipid accumulation in hepatocytes. Furthermore, H19‐induced lipid accumulation was effectively inhibited by PI3K/mTOR inhibitor PF‐04691502. Accordingly, H19 overexpression in hepatocytes up‐regulated most components of the mTORC1 signalling axis, which were inhibited by silencing endogenous H19. In vivo hepatocyte implantation studies further confirm that H19 promoted hepatic steatosis by up‐regulating both mTORC1 signalling axis and MLXIPL transcriptional network. Collectively, these findings strongly suggest that H19 may play an important role in regulating hepatic lipid metabolism and may serve as a potential therapeutic target for NAFLD.

## 1 INTRODUCTION

Liver has numerous critical metabolic functions that include metabolism of nutrients, synthesis of glucose and lipids, and detoxification of drugs and xenobiotics.^1^, ^2^, ^3^, ^4^ Disruptions of liver metabolic homeostasis may lead to a broad range of liver diseases, from metabolic syndrome to cancer.^1^, ^2^, ^3^, ^4^ The rapid increase in obesity and the metabolic syndrome worldwide exacerbates the pathological changes that lead to non‐alcoholic fatty liver disease (NAFLD), which is the most common liver disease in the Western world.^2^, ^3^, ^4^ The hallmark of NAFLD is the accumulation of triglycerides within hepatocytes (also known as, hepatic steatosis), which is strongly associated with obesity and the metabolic syndrome.^1^, ^2^, ^3^, ^4^

Hepatic lipid metabolism is tightly regulated through a delicate interplay of hormones (such as insulin), nuclear receptors (such as LXR), numerous cellular signalling pathways (such as PI3K/AKT/mTOR) and hepatic transcription factors (such as MLXIPL/ChREBP and SREBPs).^1^, ^5^, ^6^, ^7^, ^8^, ^9^, ^10^, ^11^ Nonetheless, detailed molecular mechanisms through which hepatic lipid metabolism is regulated remain to be fully understood. Emerging evidence suggests that non‐coding RNAs (ncRNAs), especially long non‐coding RNAs (lncRNAs), may play an important role in liver metabolism and metabolic diseases.^12^, ^13^, ^14^, ^15^

In this study, we investigated the role of lncRNA H19 in regulating hepatic lipid metabolism. H19 was identified as one of the first ncRNAs nearly three decades ago.^16^, ^17^ H19 is located in a highly conserved imprinted gene cluster containing a neighbouring reciprocally imprinted gene for *Igf2*.^18^ While *Igf2* is paternally expressed, H19 for both human and mouse is expressed from the maternal allele. H19 expression is strongly induced during embryogenesis and down‐regulated after birth, except in adult skeletal muscle and heart.^18^ Since its discovery, H19 has been shown to participate in highly diverse cellular processes, including embryonic growth, tumorigenesis, stem cell differentiation and metabolism.^19^, ^20^ We also found that H19 plays an important role in regulating BMP9‐regulated lineage‐specific differentiation of mesenchymal stem cells (MSCs).^21^ Nonetheless, the diverse biological functions of H19 remain to be fully understood.

Here, we found that H19 was up‐regulated in steatosis and high‐fat diet (HFD)‐induced NAFLD. Overexpression of H19 in hepatocytes induced lipid accumulation and up‐regulated numerous genes involved in lipid metabolism, while silencing H19 decreased lipid accumulation in hepatocytes. Mechanistically, we showed that H19 promoted hepatic steatosis by up‐regulating MLXIPL/ChREBP and silencing Mlxipl diminished H19‐induced lipid accumulation in hepatocytes. H19‐induced lipid accumulation was inhibited by PI3K/mTOR inhibitor PF‐04691502. Accordingly, H19 overexpression up‐regulated mTORC1 signalling complex in hepatocytes, which were inhibited by silencing H19. Hepatocyte implantation studies further confirmed that H19 promoted hepatic steatosis by up‐regulating both mTORC1 and MLXIPL in hepatocytes. Thus, our findings strongly suggest that H19 may play an important role in regulating hepatic lipid metabolism and may serve as a potential therapeutic target for NAFLD.

## 2 METHODS AND MATERIALS

### 2.1 Cell culture and chemicals

HEK‐293 derivatives 293pTP and RAPA cells were previously described.^22^, ^23^ Primary mouse hepatocytes were isolated from 4‐week‐old C57BL/6J mice using the type I collagenase/liver perfusion protocol as described.^24^, ^25^ Mouse hepatocyte line iHPx cells are reversibly immortalized mouse E12.5 hepatocytes as described previously.^25^, ^26^ All cell lines were maintained in complete DMEM supplemented with 10% foetal bovine serum (Lonsa Science SRL), 100 units of penicillin and 100 mg of streptomycin at 37°C in 5% CO_2_. Unless indicated otherwise, all chemicals were purchased from Sigma‐Aldrich, Thermo‐Fisher Scientific or Solarbio.

### 2.2 Establishment of the mouse model of non‐alcoholic fatty liver disease (NAFLD)

The use and care of experimental animals was approved by the Research Ethics and Regulations Committee of Chongqing Medical University (Chongqing, China; permit no: SCXK(YU)20070001). All animal experiments were performed in accordance with US National Institutes of Health Guide for the Care and Use of Labotatory animals.^27^ The mouse model of NAFLD was established as previously described.^28^ Briefly, 60 mice (C57BL/6, male, interpretation period) were obtained from and housed in the Experimental Animal Research Center at Chongqing Medical University. The mice were randomly divided into two groups, the high‐fat diet (HFD) group and the control group (30 each). The HFD group was fed with the 45% fat diet (Medicience), whereas the control group was fed with 10% fat diet (Table S1). Ten mice from each group were sacrificed at week 10, week 16 and week 24, respectively. The retrieved liver tissue was either fixed with 4% paraformaldehyde for histologic evaluation and immunostaining or snap‐frozen in liquid nitrogen for total RNA/protein isolation.

### 2.3 Construction and amplification of recombinant adenoviruses

Recombinant adenoviruses were generated by using the AdEasy technology.^29^, ^30^, ^31^ Briefly, the full‐length transcript of mouse H19 was PCR amplified for generating recombinant adenoviral plasmid pAd‐H19 as described.^21^ Recombinant adenovirus Ad‐H19 was produced and/or amplified in 293pTP or RAPA cells. The resulting Ad‐H19 adenovirus also co‐expresses RFP as a marker for tracking infection efficiency. For constructing the adenoviral vectors expressing siRNAs targeting mouse H19 and mouse Mlxipl/ChREBP, we employed our recently developed pSOS system, in which siRNA expression is driven by the converging U6‐H1 promoters.^32^, ^33^, ^34^, ^35^ The siRNA sites targeting mouse H19 or Mlxipl were designed by using Invitrogen's BLOCK‐iTRNAi Designer and/or Dharmacon Horizon Discovery's siDESIGN programs (Table S2). The oligo cassettes containing the siRNA sites were cloned into an adenoviral shuttle vector, followed by homologous recombination in BJ5183 cells as described.^21^, ^29^ The resulting recombinant adenoviral vectors were used to generate adenovirus Ad‐siH19 or Ad‐siMlxipl, each of which contains the pooled three siRNAs, and also co‐expresses the RFP marker gene. Adenoviral vector expresses RFP (Ad‐RFP) alone was used as a mock adenovirus control. For all adenoviral infections, polybrene (8 µg/mL) was added to enhance infection efficiency as described.^36^

### 2.4 Oil Red O (ORO) staining for triglyceride/lipid accumulation

The ORO staining was carried out as described.^37^ Briefly, primary mouse hepatocytes were seeded in 24‐well culture plates and treated with different conditions for 10 days. Alternatively, frozen sections from freshly prepared liver tissue or subcutaneous transplantation in nude mice were washed with PBS to remove the embedding agents. Both cultured cells and frozen sections were then fixed with 4% paraformaldehyde for 10 minutes, briefly incubated in 60% isopropanol and then stained with freshly prepared ORO solution for 15 minutes, followed by PBS washes. The staining results were recorded under a bright field microscope. Each assay condition was done in triplicate.

### 2.5 Bodipy 493/503 staining (triglyceride/lipid accumulation)

Bodipy 493/503‐based fluorescent detection of lipid droplets was carried out as reported.^38^ Briefly, the primary hepatocytes were seeded in 24‐well culture plates and treated with different conditions for 10 days. Alternatively, frozen sections from freshly prepared liver tissue were washed with PBS to remove the embedding agents. Both cultured cells and frozen sections were then fixed with 4% paraformaldehyde for 10 minutes, and stained with 2 µmol/L BODIPY493/503 (4,4‐Difluoro‐1,3,5,7,8‐Pentamethyl‐4‐Bora‐3a,4a‐Diaza‐s‐Indacene; Sigma‐Aldrich) for 15 minutes, followed by PBS washes. The staining results were recorded under a fluorescence microscope. Each assay condition was done in triplicate.

### 2.6 Total RNA isolation and touchdown‐quantitative real‐time PCR (TqPCR) analysis

Total RNA from both cultured cells and freshly prepared liver tissues was isolated by using the TRIZOL Reagent (Invitrogen) by following the manufacturer's instructions. Briefly, the freshly prepared mouse liver tissue at the indicated development stages (n = 5 CD1 male mice for each time‐point) or the NAFLD model was dissected out, minced and ground in the TRIzol Reagent. Alternatively, cultured cells were lysed in TRIZOL Reagent for total RNA isolation. Total RNA was subjected to reverse transcription with hexamer and M‐MuLV reverse transcriptase (New England Biolabs). The cDNA products were used as PCR templates. The H19 RT primer and gene‐specific PCR primers were designed by using Primer3 program (Table S3). TqPCR was carried out by using 2× SYBR Green qPCR Master Mix (Bimake) on a CFX‐Connect unit (Bio‐Rad Laboratories) as described.^39^, ^40^, ^41^ All TqPCR reactions were done in triplicate. Gapdh was used as a reference gene. Quantification of gene expression was performed using the 2^−ΔΔCq^ method.^42^

### 2.7 H & E staining and immunohistochemical (IHC) staining

The retrieved mouse liver tissue was fixed in 4% paraformaldehyde overnight, paraffin embedded and sectioned. The sections were deparaffinized, rehydrated and subjected to H & E staining and IHC staining as described.^26^ Briefly, the sections were subjected to deparaffinization, followed by antigen retrieval and immunostaining with different antibodies against MLXIPL (1:100‐1:200 dilution; Gen Tex; Cat#GTX30677), p‐mTOR (phospho Ser2448 1:100‐1:200 dilution; Gene Tex; Cat#GTX132803), mTOR (1:100‐1:200 dilution; and Gene Tex; Cat#GTX101557), LIPIN1 (1:100‐1:200 dilution; Abcam; Cat#ab181389), SREBP1 antibody (1:100‐1:200 dilution; Proteintech; Cat#14099‐1‐AP). Control rabbit IgG (1:200; cat. no. 011‐000‐003; Jackson ImmunoResearch Laboratories, Inc) was used as a negative control. examined and recorded under a microscope (magnification, ×400).

### 2.8 Western blotting analysis

Western blotting assay was carried out as previously described.^43^ Briefly, subconfluent primary hepatocytes were infected with Ad‐H19, Ad‐siH19 and Ad‐RFP for 72 hours. Cell lysates were prepared and subjected to SDS‐PAGE, followed by electro‐transferring to PVDF membranes, which were blocked and incubated overnight with the primary antibodies againstβ‐ACTIN (1:5000‐1:20 000 dilution; Proteintech; Cat#60008‐1‐Ig), MLXIPL (1:1000‐1:3000 dilution; Gen Tex; Cat#GTX30677), DEPTOR (1:100 dilution; Santa Cruz Biotechnology; Cat#sc‐398169), PRAS40 (1:100 dilution; Santa Cruz Biotechnology; Cat#sc‐517355), RAPTOR (1:100 dilution; Santa Cruz Biotechnology; Cat#sc‐81537), MTOR (1:1000‐1:3000 dilution; Gene Tex; Cat#GTX101557), p‐MTOR (phospho Ser2448 1:1000‐1:3000 dilution; Gene Tex; Cat#GTX132803), S6K (1:1000‐1:3000 dilution; Gene Tex; Cat#GTX107562), p‐S6K (phosphor S424 1:1000‐1:3000 dilution; Abcam; Cat#ab131436), LIPIN1 (1:1000‐1:3000 dilution; Abcam; Cat#ab181389) and SREBP (1:1000‐1:3000 dilution; Proteintech; Cat#14099‐1‐AP). After being washed, the membranes were incubated with respective secondary antibodies (1:5000 dilution; ZSGB‐BIG; Peroxidase‐Conjugated Rabbit anti‐Goat IgG (H + L) or Peroxidase‐Conjugated Goat anti‐Mouse IgG (H + L); Cat#ZB‐2306 or 2305) conjugated with horseradish peroxidase. Immune‐reactive signals were visualized by using the Enhanced Chemiluminescence (ECL) kit (Millipore) on the Bio‐Rad ChemiDoc Imager.

### 2.9 Immunofluorescence staining

Immunofluorescence staining assay was carried out as previously described.^21^ The primary hepatocytes were treated with adenoviral infection and fixed with 4% paraformaldehyde. The fixed cells were then treated with 0.1% Triton‐100 and blocked with 10% bovine serum albumin, followed by incubated with MLXIPL antibody (1:100‐1:200 dilution; Gen Tex; Cat#GTX30677) in 4°C overnight and stained with Fluorescein (FITC)–conjugated Affinipure Goat Anti‐Rabbit IgG (H + L) (1:20‐1:100 dilution; Proteintech, Cat#SA00003‐11). Cell nuclei were counterstained with DAPI, followed by fluorescence microscopic imaging (magnification, ×400).

### 2.10 Subcutaneous hepatocyte implantation in athymic nude mice

All animal experiments were carried out in accordance with the approved guidelines approved by the Research Ethics and Regulations Committee of Chongqing Medical University. Athymic nude mice were obtained from and housed in the Experimental Animal Research Center of Chongqing Medical University. The cell implantation experiments were carried out as previously described.^44^ Briefly, subconfluent iHPx cells were infected with different adenoviruses for 36 hours, then collected and resuspended in sterile PBS for subcutaneous injection into the flanks of athymic nude mice (5‐ to 6‐week‐old, male, 5 × 10^6^ cells/injection, six injections per mouse, and five mice per group). At 10 days after injection, mice were sacrificed, and subcutaneous masses at the injection sites were retrieved for H & E staining, ORO staining and IHC staining.

### 2.11 Statistical analysis

Quantitative assays were carried out in triplicate and/or repeated three batches of independent experiments. Statistical significances were determined by one‐way analysis of variance and Student's *t* test. A value of *P* < .05 was considered statistically significant.

## 3 RESULTS

### 3.1 H19 is up‐regulated in oleic acid‐induced steatosis and during the development of a mouse model of NAFLD

**Figure 1:**
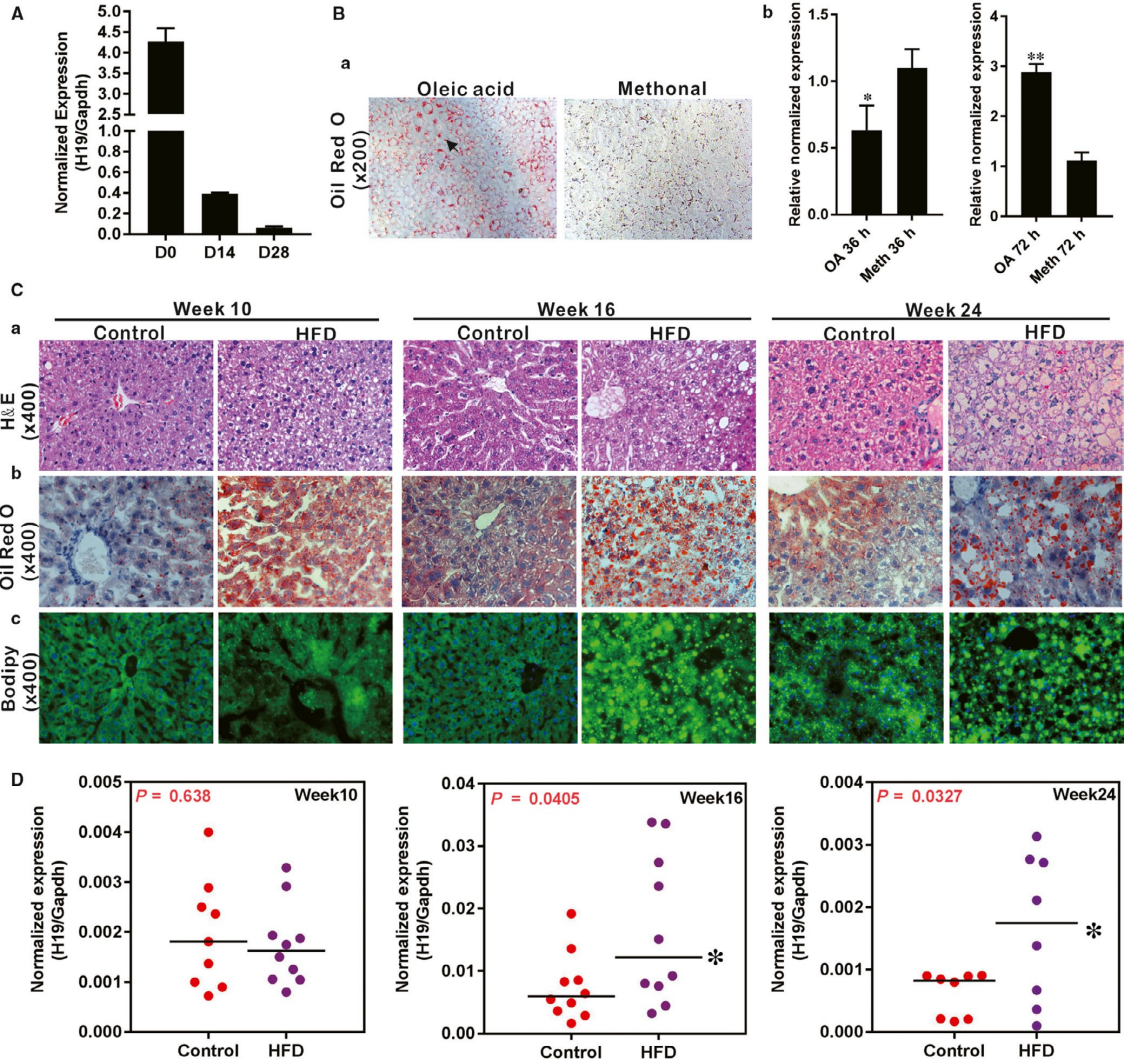
The expression of lncRNA H19 during triglyceride/lipid accumulation in hepatocytes and in a mouse model of NAFLD. A, Total RNA was isolated from mouse liver tissue at birth (D0), 14 d (D14) and 28 d (D28) after birth. TqPCR analysis was carried out to assess H19 expression. B, Primary mouse hepatocytes were stimulated with 0.05 mmol/L oleic acid (OA, methanol as the solvent control) for the indicated time‐points and subjected to the Oil Red O (ORO) staining after 3 d (a). TqPCR analysis of H19 expression at 36 and 72 h during triglyceride/lipid accumulation in hepatocytes by OA (b). Relative expression ratio was calculated by dividing the relative expression values of given genes with that of Gapdh (ie H19/Gapdh). ‘*’*P* < .05, ‘**’*P* < .01, oleic acid group vs the control (methanol) group. C, Establishment of a mouse model of HFD‐induced NAFLD. C57/B6 mice (male, n = 10/time‐point/group) were fed with 45% high‐fat diet (HDF) or normal diet (Control), and sacrificed at weeks 10, 16 and 24, respectively. The liver tissue was retrieved and subjected to H & E staining (a), ORO staining (b) and Bodipy 493/503 staining (c). D, The expression of H19 during development of NAFLD. Total RNA was isolated from the mouse liver tissue of the HFD and Control groups at weeks 10, 16 and 24 respectively, and subjected to TqPCR analysis of H19 expression. Relative expression ratio was calculated by dividing the relative expression values of H19 with that of Gapdh (ie H19/Gapdh). ‘*’*P* < .05, HFD group vs Control diet group

We first analysed the H19 expression during post‐natal liver development and found that H19 expression was extremely high in newborn liver, but drastically decreased in 2‐week and 4‐week liver tissue (Figure 1A). We next examined the H19 expression status during hepatic steatosis and found that during oleic acid (OA, at 0.05 mmol/L)‐induced hepatic steatosis,^45^ H19 expression was significantly up‐regulated in hepatocytes at 72 hours after OA treatment (Figure 1B, panels a and b), suggesting that H19 expression may be associated with hepatic steatosis.

We further analysed H19 expression during the development of a mouse model of HFD‐induced NAFLD. As shown in (Figure 1C), we successfully established the mouse model of NAFLD.^28^ Specifically, H & E evaluation indicates that the liver tissue retrieved from the HFD treatment groups exhibited noticeable lipid accumulation at week 10, significantly increased lipid accumulation at week 16 and a full‐blown steatosis phenotype at week 24, compared with the control groups (Figure 1C, panel a). The HFD‐induced temporal lipid accumulations were further confirmed by ORO staining and Bodipy 493/503 staining (Figure 1C, panels b and c). Interestingly, qPCR analysis revealed that H19 expression in NAFLD liver tissue significantly elevated at week 16 and week 24, when compared with that of the control groups (Figure 1D). These results suggest that lncRNA H19 expression may be associated with the development of hepatic steatosis.

### 3.2 Exogenous H19 induces triglyceride/lipid accumulation, while silencing endogenous H19 leads to decreased lipid accumulation in hepatocytes

**Figure 2:**
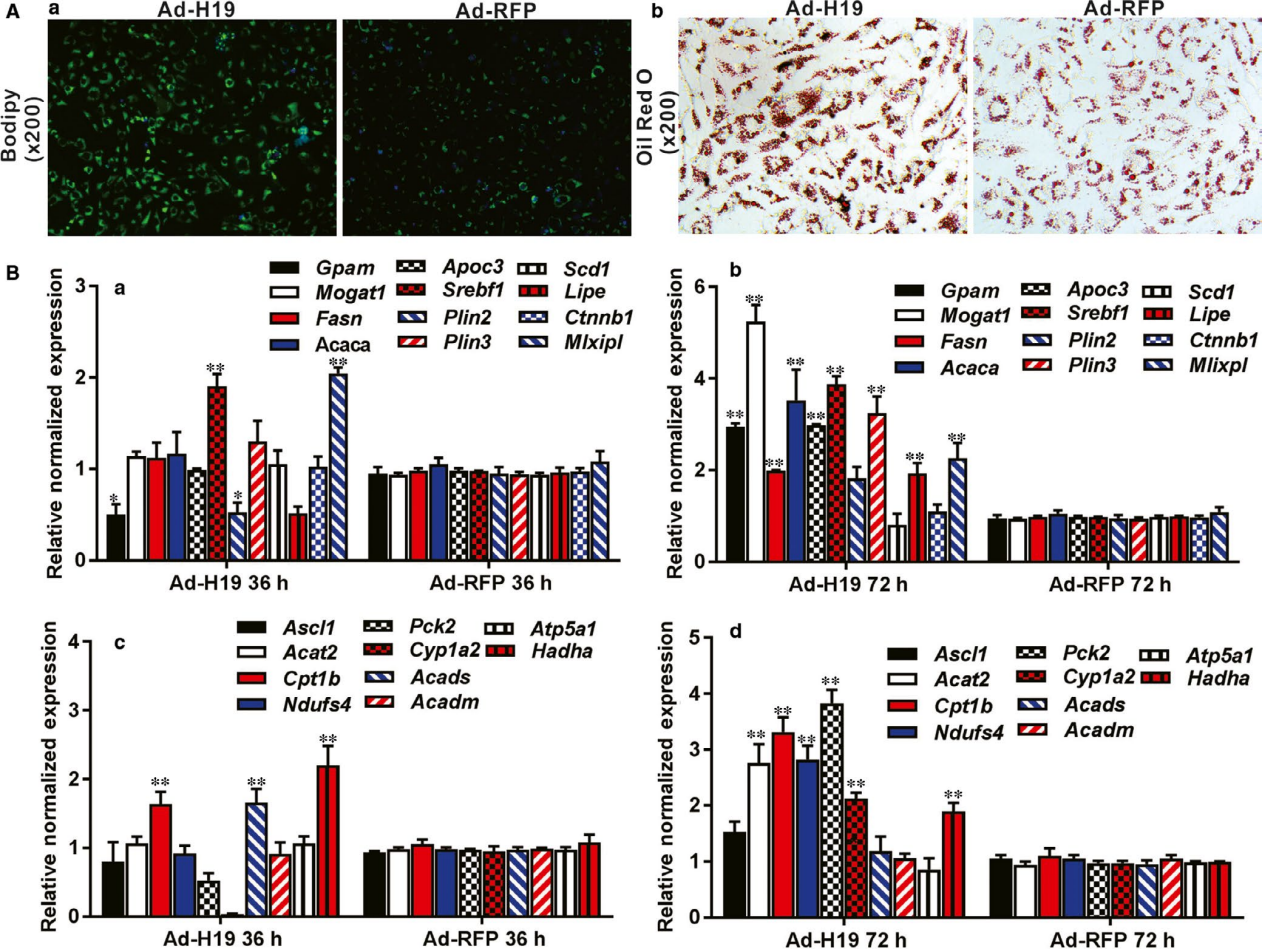
Exogenously expressed H19 induces triglyceride/lipid accumulation and regulates the expression of the genes involved in lipid metabolism in hepatocytes. A, Overexpression of H19 induces lipid accumulation in hepatocytes. Primary mouse hepatocytes were infected with Ad‐H19 or Ad‐RFP for 7 d, and subjected to Bodipy 493/503 staining (a) and ORO staining (b). Each assay condition was done in triplicate. Representative images are shown. B, H19 regulates the expression of the genes involved in triglyceride synthesis, storage and breakdown in hepatocytes. Primary mouse hepatocytes were infected with Ad‐H19 or Ad‐RFP for 36 and 72 h. Total RNA was isolated and subjected to TqPCR analysis of the expression of the triglyceride synthesis and storage‐related genes (a and b) and triglyceride breakdown‐related genes (c and d). Relative expression ratio was calculated by dividing the relative expression values of given genes with that of Gapdh (ie gene/Gapdh). ‘*’*P* < .05, ‘**’*P* < .01, Ad‐H19 group vs Ad‐RFP group

We examined whether altered H19 expression would directly affect hepatic lipid metabolism. Using the adenoviral vector Ad‐H19 that overexpresses mouse H19 (Figure S1A panel a), we found that H19 overexpression resulted in significantly increased lipid accumulation in hepatocytes as revealed by both Bodipy 493/503 staining (Figure 2A panel a) and ORO staining (Figure 2A panel b) compared with the RFP control. Furthermore, qPCR analysis revealed that H19 overexpression in hepatocytes led to the increased expression of numerous genes involved in lipid synthesis and storage, such as Gpam, Mogat, Fasn, Acaca, Apoc3, Srebp, Plin2, Plin3, Lipe and Mlxipl at 72 hours after Ad‐H19 infection (Figure 2B panels a and b). Interestingly, forced H19 expression also up‐regulated several genes involved in lipid breakdown, such as Acat2, Cpt1b, Ndafs4, Pck2, Cyp1a2 and Hadha at 72 hours after Ad‐H19 infection, although Pck2 and Cyp1a2 were down‐regulated while Cpt1b, Acads and Hadha were up‐regulated at 36 hours after infection (Figure 2B panels c and d). These results indicate that the lipid synthesis and storage‐related genes and lipid breakdown‐related genes may be tightly regulated in a coordinated fashion so that exogenous H19 expression would simultaneously affect the expression of both pathways of lipid metabolism.

**Figure 3:**
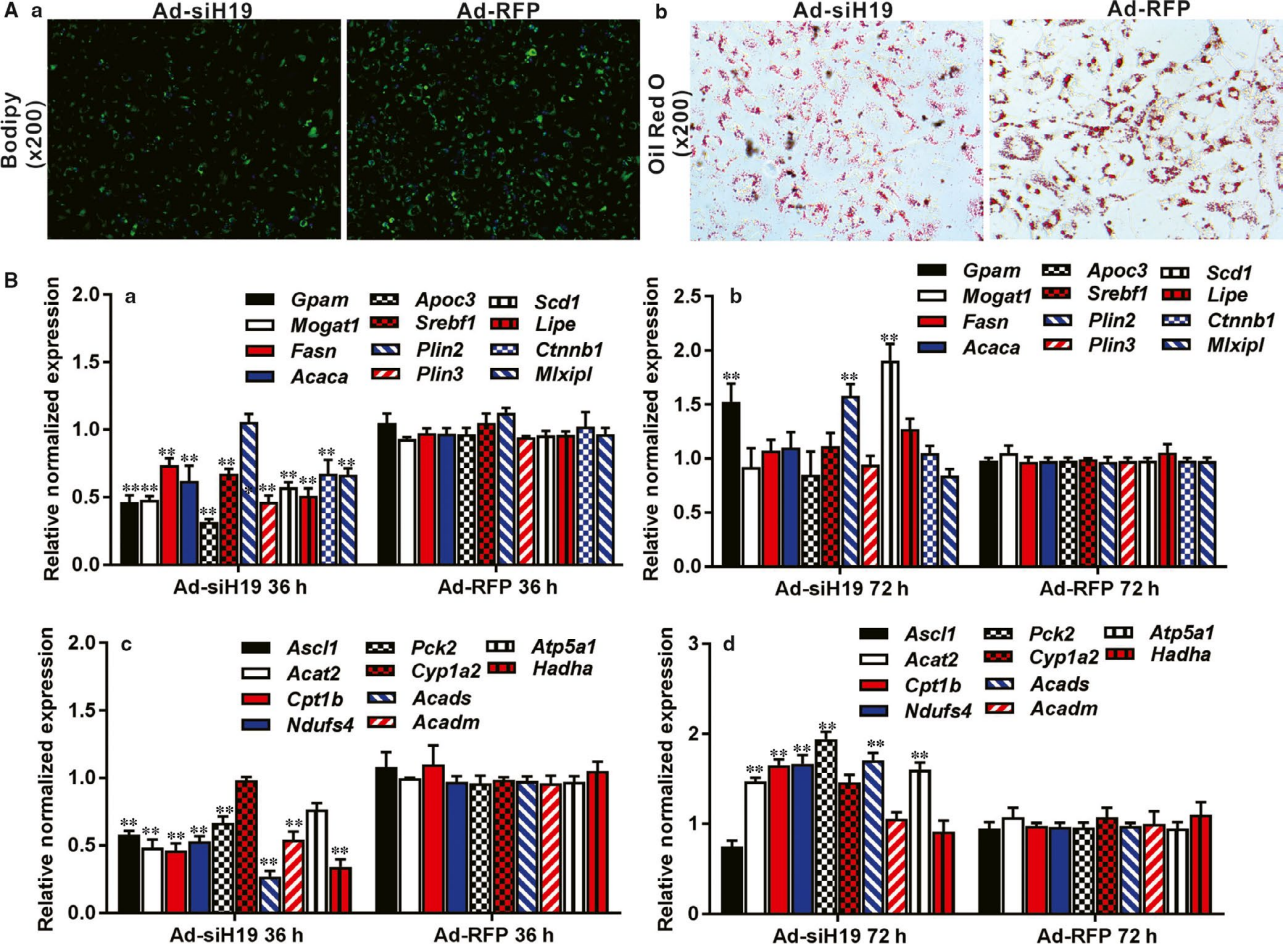
Silencing endogenous H19 expression inhibits triglyceride/lipid accumulation and down‐regulates the expression of the genes that are involved in lipid metabolism. A, Silencing H19 expression inhibits endogenous hepatic triglyceride/lipid accumulation. Primary mouse hepatocytes were infected with Ad‐siH19 or Ad‐RFP for 10 d, and subjected to Bodipy493/503 staining (a) and ORO staining (b). Each assay condition was done in triplicate. Representative images are shown. B, Silencing H19 expression inhibits the expression of the genes involved in lipid metabolism in hepatocytes. Primary mouse hepatocytes were infected with Ad‐siH19 or Ad‐RFP for 36 and 72 h. Total RNA was isolated and subjected to TqPCR analysis to detect the expression of the triglyceride synthesis and storage‐related genes (a and b) and triglyceride breakdown‐related genes (c and d). All samples were normalized with Gapdh. Each assay condition was done in triplicate. Relative expression ratio was calculated by dividing the relative expression values of given genes with that of Gapdh (ie gene/Gapdh). ‘**’*P* < .01, Ad‐siH19 group vs Ad‐RFP group

We further analysed the effect of silencing endogenous H19 on hepatic lipid metabolism. In order to effectively silence mouse H19, we constructed an adenoviral vector, Ad‐siH19, to express three siRNAs that target mouse H19 (Figure S1A panel b), and found that silencing endogenous H19 decreased the basal level of lipid accumulation in hepatocytes as revealed by both Bodipy493/503 staining (Figure 3A panel a) and ORO staining (Figure 3B panel b), compared with the RFP control. Quantitative expression analysis revealed that silencing H19 significantly inhibited the expression of most of the genes involved in lipid synthesis and storage, such as Gpam, Mogat, Fasn, Acaca, Apoc3, Srebp, Plin3, Lipe, Ctnnb1 and Mlxipl, at 36 hours after Ad‐siH19 infection although elevated expression of Gpam, Plin2 and Scd1 was found at 72 hours after Ad‐siH19 infection (Figure 3B panels a and b). Similar to H19 overexpression though, silencing H19 expression also up‐regulated most of the genes involved in lipid breakdown, such as Acat2, Cpt1b, Ndafs4, Pck2, Cyp1a2, Acads and Atpsa1, at 72 hours after Ad‐H19 infection, although most of them were down‐regulated at 36 hours after infection (Figure 3B panels c and d). Taken together, these results suggest that H19 may directly regulate lipid metabolism in hepatocytes.

### 3.3 H19 promotes hepatic steatosis by up‐regulating MLXIPL, while silencing Mlxipl diminishes H19‐induced lipid accumulation in hepatocytes

**Figure 4:**
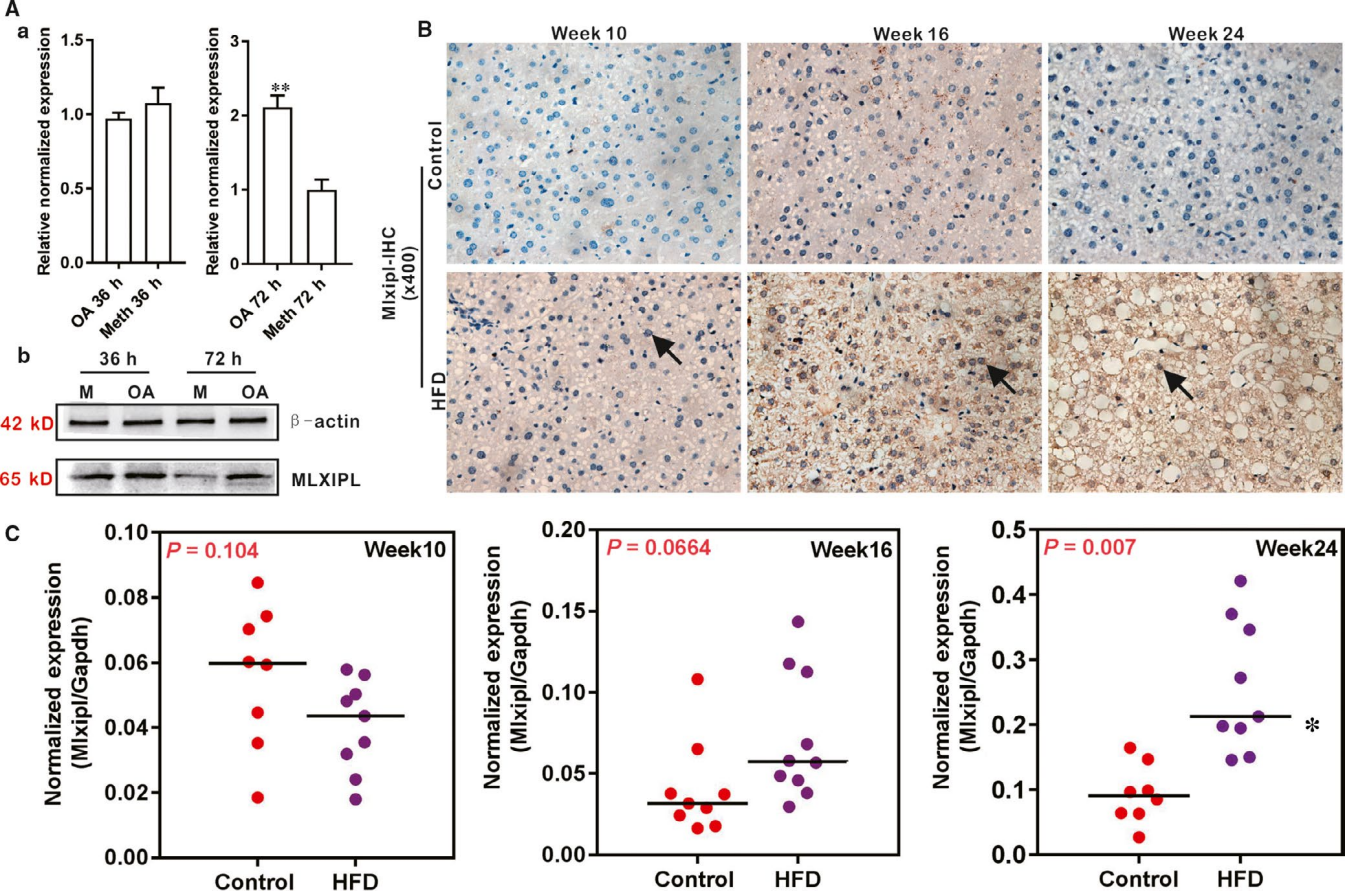
The expression of MLXIPL is up‐regulated during triglyceride/lipid accumulation in hepatocytes and in a mouse model of NAFLD. A, MLXIPL is up‐regulated during oleic acid‐induced triglyceride/lipid accumulation in hepatocytes. Primary mouse hepatocytes were stimulated with 0.05 mmol/L oleic acid (methanol as the solvent control) for the indicated time‐points and total RNA was isolated and subjected to qPCR (a) or total cell lysate was prepared for Western blotting analysis (b) to assess MLXIPL expression at 36 and 72 h. Each assay condition was done in triplicate. Relative expression ratio was calculated by dividing the relative expression values of given genes with that of Gapdh (ie Mlxipl/Gapdh). ‘**’*P* < .01, oleic acid group vs the control (methanol) group. B, Immunohistochemical (IHC) staining of MLXIPL expression in liver tissue retrieved from HFD‐induced NAFLD. Sections of the liver tissue retrieved from the HFD and the control groups were subjected to IHC staining using an anti‐MLXIPL antibody or IgG control. Positive nuclear stains of MLXIPL expression are indicated by arrows. Representative images are shown. C, Up‐regulated expression of Mlxipl in HFD induced NAFLD. Total RNA was isolated from the mouse liver tissue of the HFD and Control groups at weeks 10, 16, and 24 respectively, and subjected to TqPCR analysis of Mlxipl expression. Each assay condition was done in triplicate. Relative expression ratio was calculated by dividing the relative expression values of Mlxipl with that of Gapdh (ie Mlxipl/Gapdh). ‘*’*P* < .05, the HFD group vs Control diet group

To explore potential mechanism underlying the regulatory role of H19 in hepatic lipid metabolism, we examined whether the glucose‐dependent lipogenic transcription factor MLXIPL was involved in H19‐regulated hepatic lipid metabolism. We first confirmed that MLXIPL expression was significantly up‐regulated in OA‐induced hepatic steatosis both at mRNA and protein levels (Figure 4A panels a and b). We also analysed the MLXIPL expression in the liver tissue of HFD‐induced NAFLD and found that MLXIPL expression was significantly elevated at weeks 16 and 24 of NAFLD development at both protein (Figure 4B) and mRNA (Figure 4C) levels. Control IgG of liver tissue was shown (Figure S1B panel a). Collectively, these results indicate that MLXIPL expression is strongly associated with hepatic steatosis.

**Figure 5:**
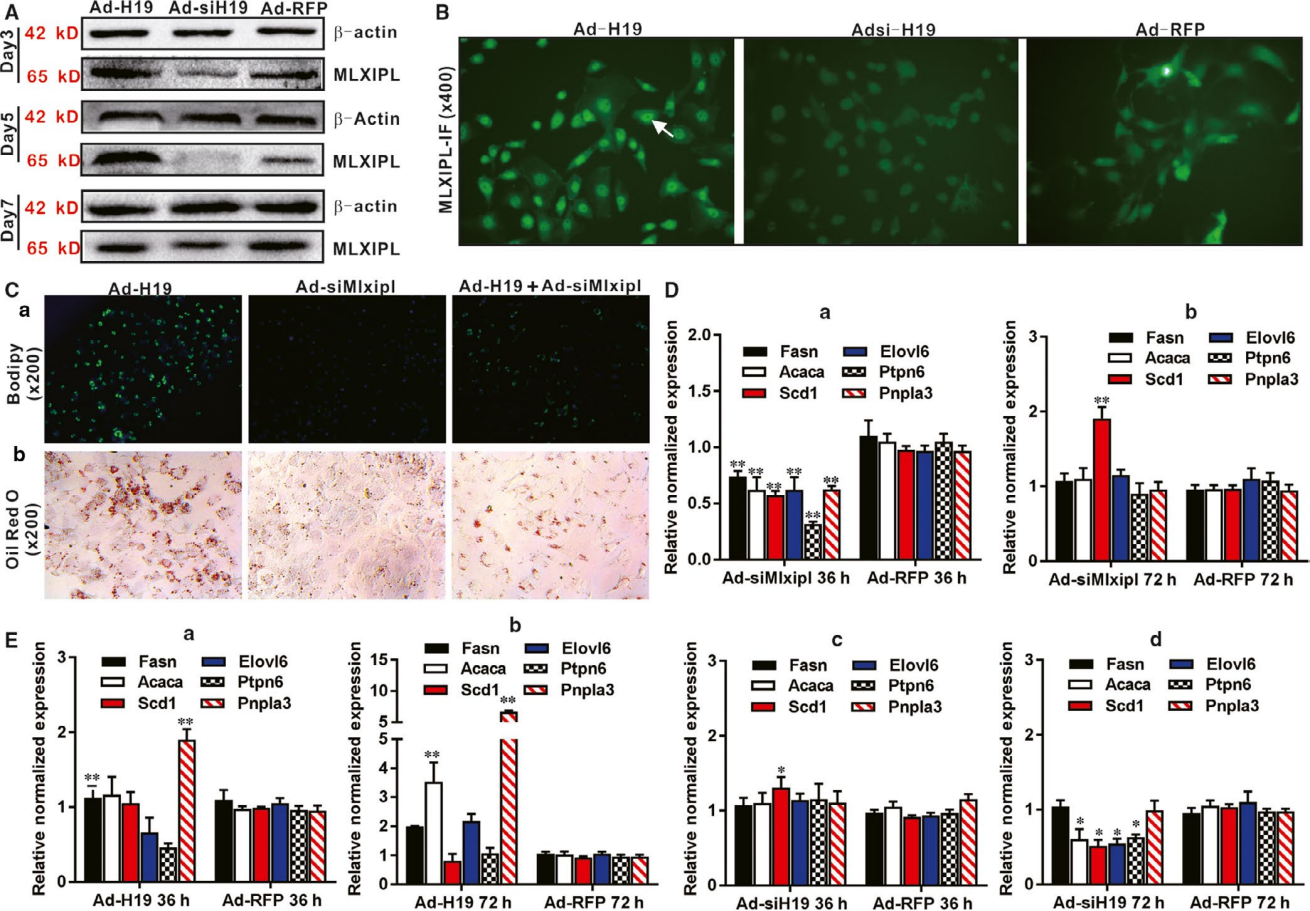
H19 induces lipid accumulation through regulating MLXIPL in hepatocytes. A, Western blotting analysis of the effect of H19 on MLXIPL expression. Primary mouse hepatocytes were infected with Ad‐H19, Ad‐siH19 or Ad‐RFP for 3, 5 and 7 d. Total cell lysate was collected and subjected to Western blotting analysis with antibody against MLXIPL or β‐actin, which was used as a loading control. B, H19 up‐regulates the expression of MLXIPL and promotes MLXIPL transfer to the nucleus. Primary mouse hepatocytes were infected with Ad‐H19, Ad‐siH19 or Ad‐RFP for 72 h, and subjected to IF staining using an anti‐MLXIPL antibody or IgG control. Positive nuclear stains of MLXIPL expression are indicated by arrows. Representative images are shown. C, Silencing Mlxipl expression suppresses H19‐induced lipid accumulation. Primary mouse hepatocytes were co‐infected with Ad‐H19, Ad‐siMlxipl, Ad‐H19 + Ad‐siMlxipl for 7 d, and subjected to Bodipy 493/503 staining and ORO staining. Representative images are shown. D, Silencing Mlxipl suppresses the expression of Mlxipl downstream target genes. Primary mouse hepatocytes were infected with Ad‐siMlxipl and Ad‐RFP for 36 h (a) and 72 h (b), respectively. Total RNA was isolated and subjected to TqPCR analysis of the expression of Mlxipl target genes. All samples were normalized with Gapdh. Each assay condition was done in triplicate. Relative expression ratio was calculated by dividing the relative expression values of individual genes with that of Gapdh (ie gene/Gapdh). ‘**’*P* < .01, treatment group vs Ad‐RFP group. E, H19 expression impacts the expression of Mlxipl target genes. Primary mouse hepatocytes were infected with Ad‐H19, Ad‐siH19 or Ad‐RFP for 36 h (a and c) and 72 h (b and d), respectively. Total RNA was isolated and subjected to TqPCR analysis of the expression of Mlxipl target genes. All samples were normalized with Gapdh. Each assay condition was done in triplicate. Relative expression ratio was calculated by dividing the relative expression values of individual genes with that of Gapdh (ie gene/Gapdh). ‘*’*P* < .05, ‘**’*P* < .01, treatment group vs Ad‐RFP group

We next analysed whether MLXIPL expression would be regulated by H19. We found that Ad‐H19‐mediated H19 overexpression in hepatocytes increased MLXIPL expression at 3 and 5 days after infection, while silencing H19 significantly inhibited MLXIPL expression in hepatocytes at protein level (Figure 5A). Immunofluorescence staining indicated that H19 overexpression increased MLXIPL expression and nuclear translocation whereas silencing H19 expression significantly inhibited MLXIPL expression and nuclear translocation in hepatocytes (Figure 5B).

We further analysed the essential role of Mlxipl in H19‐promoted hepatic steatosis by silencing endogenous Mlxipl expression. Using the adenoviral vector Ad‐siMlxipl that expresses three siRNAs targeting mouse Mlxipl (Figure S1A panel c), we found that H19‐induced lipid accumulation was effectively inhibited by silencing Mlxipl expression in hepatocytes as revealed by Bodipy 493/503 staining and ORO staining (Figure 5C panels a and b). Furthermore, we examined the effect of H19 expression on Mlxipl‐regulated target genes. As expected, silencing Mlxipl effectively down‐regulated the expression of its target genes including Fasn, Acaca, Elovl6, Scd1, Ptpn6 and Pnpla3 at 36 hours after Ad‐siMlxipl infection although their expression was restored to the control level at 72 hours (Figure 5D panels a and b). Conversely, H19 overexpression was shown to up‐regulate the expression of three of the six tested Mlxipl target genes (eg Fasn, Acaca and Pnpla3) in hepatocytes (Figure 5E panels a and b). However, silencing H19 prevented the up‐regulation of four of the six Mlxipl target genes, for example, Acaca, Elovl6, Scd1 and Ptpn6, at 72 hours (Figure 5E panels c and d). Taken together, these results suggest that MLXIPL may be an important mediator of H19‐promoted hepatic steatosis.

### 3.4 H19 regulates hepatic lipid metabolism through activating the mTORC1 signalling axis

**Figure 6:**
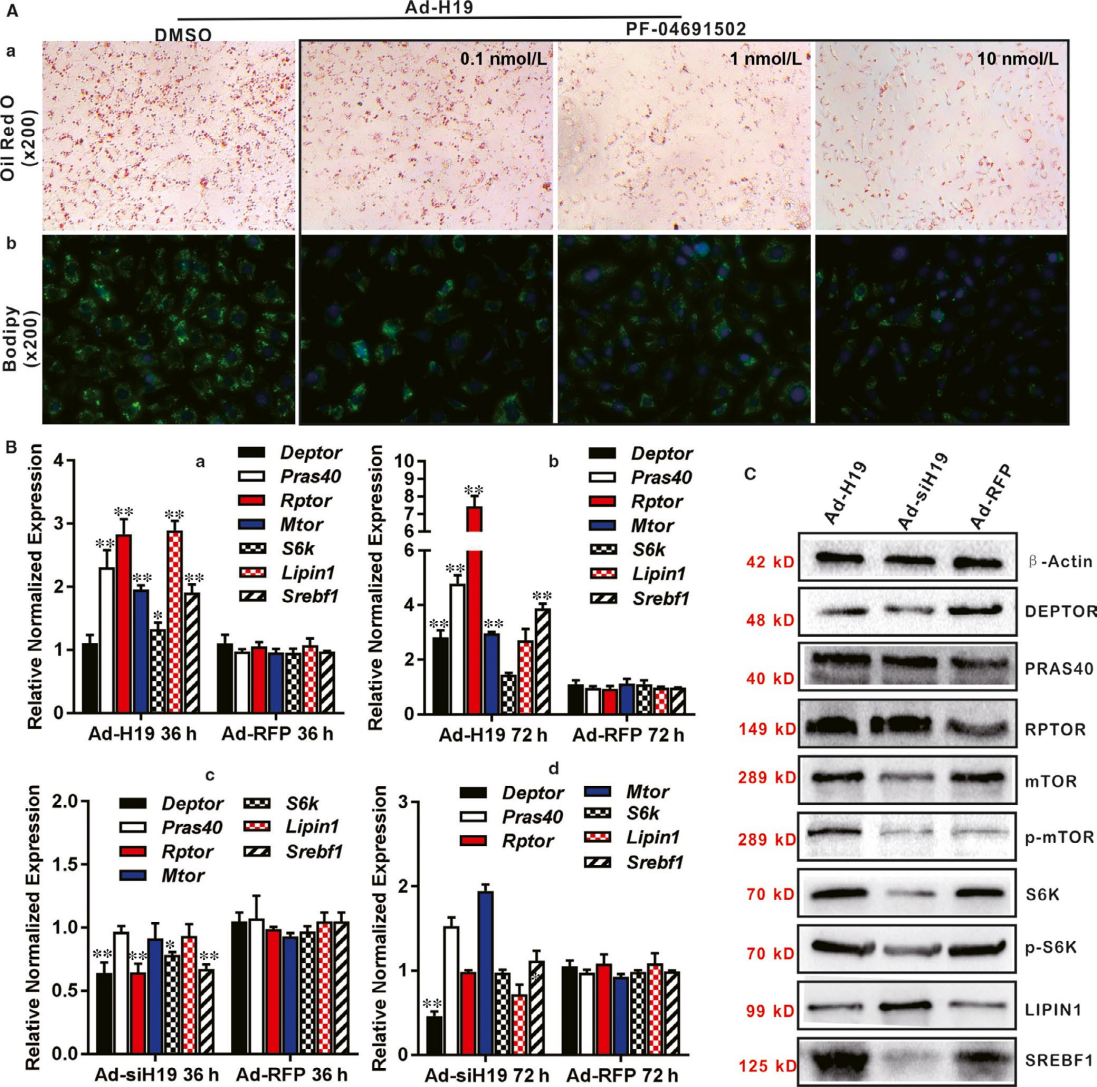
H19 regulates lipid metabolism through activating mTORC1 signalling axis. A, Blockade of PI3K/mTOR diminishes H19‐induced triglyceride/lipid accumulation in hepatocytes. Primary mouse hepatocytes were infected with Ad‐H19 for 36 h and treated with different concentrations of the PI3K/mTOR inhibitor PF‐04691502, or DMSO for 5 d, and then subjected to ORO staining (a) and Bodipy 493/503 staining (b). Representative images are shown. B, TqPCR analysis of H19‐regulated expression of members of the mTORC1 pathway. Primary mouse hepatocytes were infected with Ad‐H19, Ad‐siH19 or Ad‐RFP for 36 and 72 h, respectively. Total RNA was isolated for TqPCR analysis of expression of the members of mTORC1 signalling pathway in H19 over‐expressing hepatocytes (a and b) or H19 silenced cells (c and d). Relative expression ratio was calculated by dividing the relative expression values (ie gene/Gapdh). ‘*’*P* < .05, ‘**’*P* < .01, Ad‐H19 or Ad‐siH19 group vs Ad‐RFP group. C, Western blotting analysis of the effect of H19 on the expression of the components of mTORC1 pathway. Primary mouse hepatocytes were infected with Ad‐H19, Ad‐siH19 or Ad‐RFP for 72 h. Total cell lysate was collected and subjected to Western blotting analysis with antibodies against members of the mTORC1 pathway. Levels of β‐Actin were used as loading controls

Considering the important role of mTORC1 in lipid metabolism, we also analysed whether the mTORC1 signalling was involved in H19‐induced hepatic steatosis. Using the PI3K/mTOR dual inhibitor PF‐04691502, we found that H19‐induced lipid accumulation was effectively inhibited by PF‐04691502 in a dose‐dependent manner as revealed by ORO staining and Bodipy 493/503 staining (Figure 6A, panels a and b). Accordingly, H19 overexpression up‐regulated most components of the mTOR signalling complex, including Deptor, Pras40, Raptor, Mtor, S6k, Lipin1 and Srebp in hepatocytes (Figure 6B panels a and b). Conversely, silencing endogenous H19 inhibited and/or prevented the expression of these genes at 36 or 72 hours after Ad‐siH19 infection, except that Pras40 and Mtor were up‐regulated at 72 hours (Figure 6B panels c and d). The effect of H19 overexpression or knockdown on mTORC1 signalling complex was further analysed by Western blotting, and we found that mTOR, p‐mTOR, S6K, p‐S6K and SREBP were significantly up‐regulated by H19 overexpression, while LIPIN1 was down‐regulated at the protein levels (Figure 6C). Collectively, these results strongly suggest that mTORC1 signalling complex may be implicated in H19‐promoted hepatic lipid accumulation.

### 3.5 H19 promotes hepatic steatosis by up‐regulating both MLXIPL and mTORC1 signalling networks in hepatocyte transplantation assay

**Figure 7:**
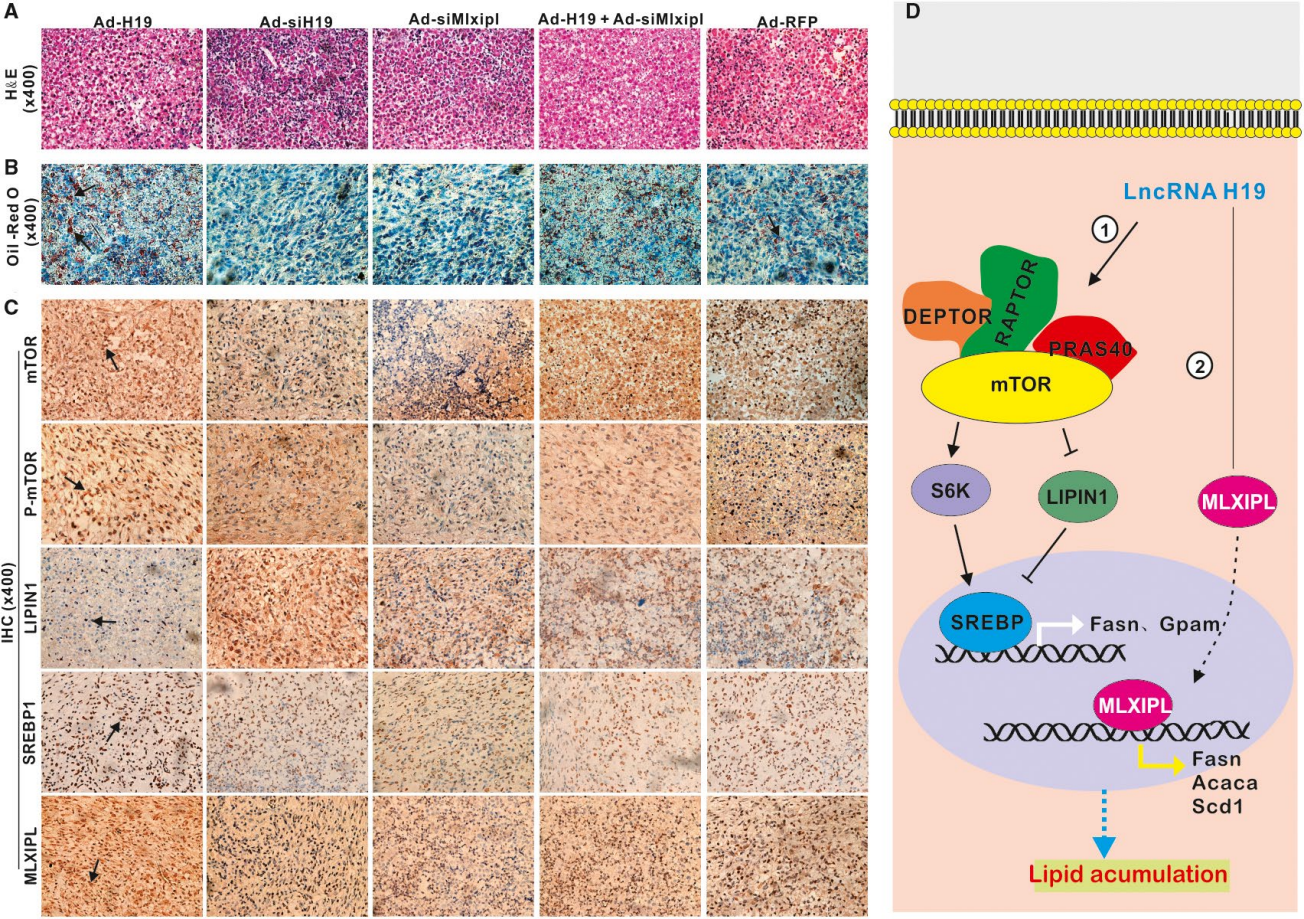
H19 promotes lipid accumulation by up‐regulating mTORC1 and MLXIPL signalling pathways in hepatocyte transplantation assay. Subconfluent mouse hepatocyte iHPx cells were infected with Ad‐H19, Ad‐siH19, Ad‐siMlxipl, Ad‐H19 + Ad‐siMlxip or Ad‐RFP for 36 h. The infected hepatocytes were collected and subcutaneously injected into the flanks of athymic nude mice for 10 d. Masses at the injection sites were retrieved, fixed and snap‐frozen or paraffin embedded. A, Paraffin sections of the retrieved cell masses were subjected to H & E staining. B, Frozen sections were subjected to ORO staining. C, Paraffin sections were further subjected to IHC staining with antibodies against mTOR, p‐mTOR, LIPIN1, SREBP and MLXIPL. Stains without primary antibody were used as negative controls. Representative images are shown while representative positive stains are indicated by arrows. D, A working model of action for LncRNA H19 in regulating hepatic steatosis. H19 may regulate hepatic lipid metabolism through at least two pathways, the mTOR signalling axis and Mlxipl‐regulated transcription network in hepatocytes

Even although mouse H19 has bene knocked out,^46^, ^47^ it remains challenging to directly assess the effect of H19 on hepatocytes. Here, we analysed the effect of H19 on hepatic lipid metabolism in subcutaneously implanted hepatocytes in vivo. When iHPx hepatocyte line was infected with Ad‐H19, Ad‐siH19, Ad‐siMlxipl, Ad‐H19 + Ad‐siMlxipl or Ad‐RFP and implanted subcutaneously into the flanks of athymic mice for 10 days, H & E staining of the retrieved masses revealed an apparent lipid accumulation in the Ad‐H19 group, compared with the Ad‐RFP control group, which was inhibited by silencing H19 expression (Figure 7A). ORO staining confirmed the H & E staining results and showed that H19 overexpression led to lipid accumulation, which was effectively inhibited by H19 knockdown (Figure 7B). IHC staining revealed that H19 overexpression significantly up‐regulated the expression of mTOR and p‐mTOR, and the nuclear localization of SREBP and MLXIPL proteins, which were attenuated by H19 knockdown, and down‐regulated the expression of LIPIN1 (Figure 7C). Control IgG of masses was shown (Figure S1B panel b).Collectively, these results strongly suggest that lncRNA H19 may promote hepatic steatosis by up‐regulating both mTORC1 signalling complex and MLXIPL transcriptional network in hepatocytes (Figure 7D).

## 4 DISCUSSION

Even though lncRNA H19 represents one of the earliest identified non‐coding RNAs,^16^, ^17^ the exact biological functions of H19 remain to be fully understood. In this study, we investigated potential role of H19 in hepatic lipid metabolism. We found that H19 was up‐regulated in steatosis and NAFLD. Overexpression of H19 induced triglyceride/lipid accumulation and increased the expression of numerous genes involved in lipid metabolism, suggesting that the lipid synthesis and storage‐related genes and lipid breakdown‐related genes may be tightly regulated in a coordinated fashion so that exogenous H19 expression would simultaneously affect the expression of both pathways of lipid metabolism. Mechanistically, we found that H19 promoted hepatic steatosis by up‐regulating and the mTORC1 signalling axis (including active RAPTOR, mTOR, S6K SREBP and suppress LIPIN1) and MLXIPL transcriptional network in hepatocytes (Figure 7D).

Mice with targeted deletions of H19, H19d13 (deleted the gene and 10 kb upstream) and H19d3 (deleted only the 3‐kb transcription unit) were viable without apparent phenotypes.^46^, ^47^ The exact biological functions of H19 remain enigmatic, which is further complicated by complex co‐regulatory circuitry of H19‐Igf2 expression in the highly conserved imprinted gene cluster.^48^ It was reported that deletion of a nuclease‐sensitive region between the Igf2 and H19 genes led to Igf2 misregulation and increased adiposity.^49^ Liver weights of the mice lacking H19 expression markedly increased immediately after birth with significant increases in cell proliferation, possibly resulted from activating Wnt/β‐catenin and/or IGF2 signalling pathways.^50^

One important aspect of biological functions for H19 is to regulate lineage‐specific differentiation of MSCs. We found that H19 plays an important role in regulating BMP9‐regulated lineage‐specific differentiation of MSCs.^21^ It was also reported that H19 inhibited adipocyte differentiation of bone marrow MSCs through epigenetic modulation of HDACs,^51^ while a recent study suggests that H19 may regulate the balance between osteogenic and adipogenic differentiation, and enhance adipogenic differentiation through sponging miR‐188 and miR‐30a, respectively.^52^, ^53^ Furthermore, H19 was shown to promote skeletal muscle insulin sensitivity by targeting AMPK.^54^ Accordingly, H19 was shown to exhibit protective effect against dietary obesity by constraining expression of paternally expressed genes in brown fat.^55^ Consistent with our findings, H19 was shown to promote hepatic lipogenesis by regulating miR‐130a/PPARγ axis in NAFLD animal model.^56^ Moreover, a recent study suggests that H19 may serve as a lipid sensor by synergizing with the RNA‐binding polypyrimidine tract‐binding protein 1 (PTBP1) to modulate hepatic metabolic homeostasis because deletion of H19 or knockdown of PTBP1 abolished high‐fat and high‐sucrose diet‐induced steatosis.^57^ Nonetheless, it is conceivable that H19 may regulate hepatic lipid homeostasis through diverse mechanisms, especially implicating the PI3K/AKT/mTOR signalling axis. Future efforts should be devoted to delineating those possible mechanisms.

In summary, we demonstrated that lncRNA H19 expression was associated with hepatic steatosis and the development of HFD‐induced NAFLD in mice. Exogenous overexpression of H19 in hepatocytes induced triglyceride/lipid accumulation and up‐regulated the expression of numerous genes involved in lipid synthesis, storage and breakdown, while silencing endogenous H19 led to a decreased hepatic lipid accumulation. We further demonstrated that H19 achieved the above effects at least in part by up‐regulating the Mlxipl transcription network and activating the mTOR signalling axis. Collectively, our findings strongly suggest that H19 may play an important role in regulating hepatic lipid metabolism, and may serve as a potential therapeutic target for NAFLD.

## CONFLICT OF INTEREST

The authors declare that they have no competing interest.

## AUTHORS' CONTRIBUTIONS

JMF, TCH and AH conceived the project and oversaw the study; HW, YZ and QP performed most of the in vitro and in vivo experiments, and collected data; YC, LS and JMF performed H & E and IHC analyses; LR, JL, LZ, GZ, YW and QS provided essential experimental supports and important research resources; YL and JMF contributed to data analysis and statistical analysis; JMF, TCH and AH drafted the manuscript; All authors reviewed and approved the manuscript.

## Data Availability

The data used to support the findings of this study are available from the corresponding author upon request.
