# Long noncoding RNA (lncRNA) H19 induces hepatic steatosis through activating MLXIPL and mTORC1 networks in hepatocytes

**DOI:** 10.1111/jcmm.17719

**Published:** 2023-03-08

In Wang H et al,^1^ the image for ‘Ad‐siH19 of mTOR’ was mistakenly duplicated with the image from ‘Ad‐siMlxipl of p‐mTOR’ in Figure 7C. The corrected figure is shown below. The authors confirm all results and conclusions of this article remain unchanged.

**FIGURE 7:**
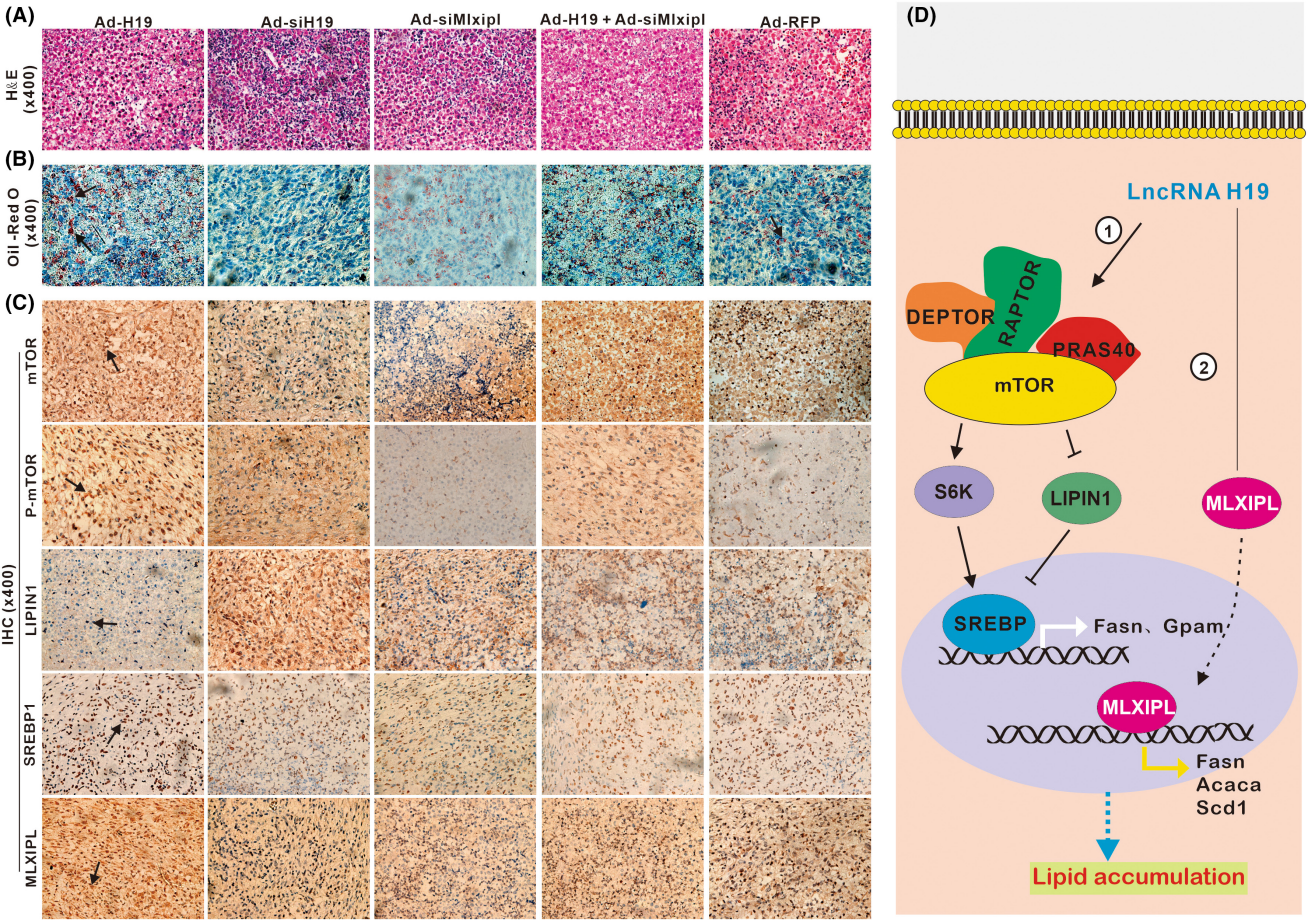
H19 promotes lipid accumulation by upregulating mTORC1 and MLXIPL signalling pathways in hepatocyte transplantation assay. Subconfluent mouse hepatocyte iHPx cells were infected with Ad‐H19, Ad‐siH19, Ad‐siMlxipl, Ad‐H19 + Ad‐siMlxip or Ad‐RFP for 36 h. The infected hepatocytes were collected and subcutaneously injected into the flanks of athymic nude mice for 10 days. Masses at the injection sites were retrieved, fixed, and snap‐frozen or paraffin‐embedded. (A) Paraffin sections of the retrieved cell masses were subjected to H&E staining. (B) Frozen sections were subjected to ORO staining. (C) Paraffin sections were further subjected to IHC staining with antibodies against mTOR, p‐mTOR, LIPIN1, SREBP and MLXIPL. Stains without primary antibody were used as negative controls. Representative images are shown while representative positive stains are indicated by arrows. (D) A working model of action for LncRNA H19 in regulating hepatic steatosis. H19 may regulate hepatic lipid metabolism through at least two pathways, the mTOR signalling axis and Mlxipl‐regulated transcription network in hepatocytes.
