# Supplementary material for: Long non‐coding RNA (lncRNA) H19 induces hepatic steatosis through activating MLXIPL and mTORC1 networks in hepatocytes
Source: J Cell Mol Med. 2019 Dec 6;24(2):1399–412. doi: 10.1111/jcmm.14818 (PMC6991647; doi:10.1111/jcmm.14818)
Supplement: Supplementary file 2 [file JCMM-24-1399-s002.doc]

**Supplemental Table 1. Formula and fatty acid composition of control (10% fat) and high fat (45% fat) diets**

|  | **MD10% Fat** | **MD45% Fat** |
| --- | --- | --- |
| **Energy Composition** | 100 | 100 |
| Protein | 20 | 20 |
| Carbohydrate | 70 | 35 |
| Fat | 10 | 45 |

| **Composition of fatty acid** | 100 | 100 |
| --- | --- | --- |
| Saturated (%) | 28.7 | 40.3 |
| Monounsaturated (%) | 32.7 | 40.4 |
| Polyunsaturated (%) | 38.6 | 19.3 |

| **Type of fat ( gm )** | 45 | 202.5 |
| --- | --- | --- |
| Lard | 20 | 177.5 |
| Soybean Oil | 25 | 25 |

| **Fatty acid profile ( gm)** | 42.2 | 190.6 |
| --- | --- | --- |
| C2, Acetic | 0 | 0 |
| C4, Butyric | 0 | 0 |
| C6, Caproic | 0 | 0 |
| C8, Caprylic | 0 | 0 |
| C10,Capric | 0.0 | 0.1 |
| C12,Lauric | 0.0 | 0.1 |
| C14,Myristic | 0.3 | 2.1 |
| C15 | 0.0 | 0.2 |
| C16,Palmitic | 7.9 | 47.1 |
| C16:1,Palmitoleic | 0.4 | 3.4 |
| C16:2 | 0 | 0 |
| C16:3 | 0 | 0 |
| C16:4 | 0 | 0 |
| C17 | 0.1 | 0.6 |
| C17:1 | 0 | 0 |
| C18,Stearic | 3.8 | 26.5 |
| C18:1,Oleic | 13.4 | 73.5 |
| C18:2,Linoleic | 14.6 | 32.4 |
| C18:3,Linolenic | 1.4 | 2.1 |
| C18:4, Stearidonic | 0 | 0 |
| C20,Arachidic | 0.0 | 0.2 |
| C20:1 | 0.0 | 0.1 |
| C20:2 | 0.1 | 0.4 |
| C20:3 | 0.0 | 1.1 |
| C20:4,Arachidonic | 0.1 | 0.7 |
| C20:5, Eicosapentaenoic | 0 | 0 |
| C21:5 | 0 | 0 |
| C22, Behenic | 0 | 0 |
| C22:1, Erucic | 0 | 0 |
| C22:4, Clupanodonic | 0 | 0 |
| C22:5,Docosapentaenoic | 0.0 | 0.2 |
| C22:6, Docosahexaenoic | 0 | 0 |
| C24, Lignoceric | 0 | 0 |
| C24:1 | 0 | 0 |
