# Supplementary material for: Long non‐coding RNA (lncRNA) H19 induces hepatic steatosis through activating MLXIPL and mTORC1 networks in hepatocytes
Source: J Cell Mol Med. 2019 Dec 6;24(2):1399–412. doi: 10.1111/jcmm.14818 (PMC6991647; doi:10.1111/jcmm.14818)
Supplement: Supplementary file 5 [file JCMM-24-1399-s005.docx]

**Supplemental Figures**

**Supplemental Fig. 1. (A)** Recombinant adenovirus-mediated overexpression of H19 or silencing of H19 and Mlxipl in mouse hepatocytes. Primary mouse hepatocytes were infected with Ad-H19 (a), Ad-siH19 (b), Ad-siMlxipl (c), or Ad-RFP. Fluorescence were observed at 36h after infection. At 48h after infection, the infected cells were collected and subjected to total RNA isolation and TqPCR analysis. Representative images are shown. Relative expression ratio was calculated by dividing the relative expression values (*i.e.*, gene/Gapdh). “**” p < 0.01, Ad-H19, Ad-siH19 or Ad-siMlxipl group *vs*. Ad-RFP group. (B) Paraffin sections of liver tissue(a) and subcutaneous masses(b) were subjected to IHC staining, stains without primary antibody were used as negative controls.
